# Five-Feature Models to Predict Preeclampsia Onset Time From Electronic Health Record Data: Development and Validation Study

**DOI:** 10.2196/48997

**Published:** 2024-08-14

**Authors:** Hailey K Ballard, Xiaotong Yang, Aditya D Mahadevan, Dominick J Lemas, Lana X Garmire

**Affiliations:** 1 Department of Computational Medicine and Bioinformatics University of Michigan Medical School Ann Arbor, MI United States; 2 Department of Health Outcomes and Biomedical Informatics University of Florida Gainesville, FL United States; 3 Center for Research in Perinatal Outcomes University of Florida Gainesville, FL United States; 4 Department of Physiology and Aging University of Florida Gainesville, FL United States; 5 Department of Obstetrics & Gynecology University of Florida Gainesville, FL United States

**Keywords:** preeclampsia, survival analysis, risk prediction, pregnancy, prognosis, survival, risk, mortality, EHR, health records, maternal, machine learning, electronic health records

## Abstract

**Background:**

Preeclampsia is a potentially fatal complication during pregnancy, characterized by high blood pressure and the presence of excessive proteins in the urine. Due to its complexity, the prediction of preeclampsia onset is often difficult and inaccurate.

**Objective:**

This study aimed to create quantitative models to predict the onset gestational age of preeclampsia using electronic health records.

**Methods:**

We retrospectively collected 1178 preeclamptic pregnancy records from the University of Michigan Health System as the discovery cohort, and 881 records from the University of Florida Health System as the validation cohort. We constructed 2 Cox-proportional hazards models: 1 baseline model using maternal and pregnancy characteristics, and the other full model with additional laboratory findings, vitals, and medications. We built the models using 80% of the discovery data, tested the remaining 20% of the discovery data, and validated with the University of Florida data. We further stratified the patients into high- and low-risk groups for preeclampsia onset risk assessment.

**Results:**

The baseline model reached Concordance indices of 0.64 and 0.61 in the 20% testing data and the validation data, respectively, while the full model increased these Concordance indices to 0.69 and 0.61, respectively. For preeclampsia diagnosed at 34 weeks, the baseline and full models had area under the curve (AUC) values of 0.65 and 0.70, and AUC values of 0.69 and 0.70 for preeclampsia diagnosed at 37 weeks, respectively. Both models contain 5 selective features, among which the number of fetuses in the pregnancy, hypertension, and parity are shared between the 2 models with similar hazard ratios and significant *P* values. In the full model, maximum diastolic blood pressure in early pregnancy was the predominant feature.

**Conclusions:**

Electronic health records data provide useful information to predict the gestational age of preeclampsia onset. Stratification of the cohorts using 5-predictor Cox-proportional hazards models provides clinicians with convenient tools to assess the onset time of preeclampsia in patients.

## Introduction

Preeclampsia is a pregnancy-associated condition characterized by new-onset hypertension and proteinuria, typically diagnosed after 20 weeks of gestation in approximately 3%-5% of all pregnancies [1]. As one of the leading causes of maternal mortality and morbidity worldwide, it can lead to a more serious condition called eclampsia if left untreated [2]. Timely identification of preeclampsia is a key factor in pregnancy risk management and subsequent treatment. Current medical practice guideline recommends prevention therapy of low-dose aspirin on women at high risk for preeclampsia before the 13-week gestation period [3]. However, preeclampsia does not typically manifest itself clinically until after 20 weeks of gestation, through clinical markers such as blood pressure (BP), urinary protein excretion, mean arterial pressure, and placental growth factor levels. Moreover, the gestational age of preeclampsia onset can vary greatly across pregnancies [3]. Preeclampsia diagnosed before 34 weeks of gestation is called early-onset preeclampsia, and late-onset preeclampsia is diagnosed after 34 weeks [4]. To allow for maximal efficiency of prevention therapy, tools that accurately predict the onset time of preeclampsia and the patient risk will be extremely beneficial.

Previous studies have identified some qualitative risk factors of preeclampsia, including preeclampsia in a previous pregnancy, a multifetal pregnancy, chronic hypertension, kidney disease, diabetes before pregnancy, autoimmune disorders, as well as demographic factors including obesity, advanced maternal age, and race [5]. However, the quantitative importance of these risk factors relative to one another has not been adequately investigated. Haile et al [6] discuss how maternal age, weight, and history of preeclampsia significantly drive preeclampsia onset time, but many additional factors remain undefined. There is an unmet need to provide clinicians with tools to accurately identify which mothers are at risk for preeclampsia, and further identify when they will develop preeclampsia.

Prognosis modeling using population-level health data provides opportunities to systematically address both issues mentioned above [7]. These new models enable the investigation of risk factors (features) that may affect the gestational age at preeclampsia diagnosis, using the hazard ratio (HR), which indicates the importance of the risk factors. Each model outputs risk factors that influence preeclampsia development and predicts the gestational age at preeclampsia diagnosis for patients using the weighted impact of each feature. In addition, patients can be stratified into low-risk and high-risk preeclampsia groups, accompanied by differences in risk factors (features). These developed and validated prognosis models will allow clinicians to practically identify when an at-risk mother might develop preeclampsia and reveal any features associated with the onset time of preeclampsia that are not included in the current guidelines.

## Methods

### Data Source

The discovery cohort for this project was obtained from the University of Michigan (UM) Medicine Healthcare System. All deidentified pregnancy records between the years 2015 and 2021, with at least one preeclampsia diagnosis, based on the *ICD-10-CM* (*International Classification of Diseases, Tenth Revision, Clinical Modification*) codes, were extracted (Table S1 in Multimedia Appendix 1). Patients who were diagnosed with competing conditions (Table S1 in Multimedia Appendix 1) were removed from the cohort. Patients who did not have any electronic medical record (EMR) in the UM system within 20 weeks of the start of their pregnancy were also removed. Since preeclampsia is clinically defined after 20 weeks, all patients with a preeclampsia diagnosis before 20 weeks of gestation were dropped from the discovery cohort. A total of 1178 pregnancies remained in the UM discovery cohort after this data selection.

Following the same inclusion and exclusion criteria, the validation cohort was generated from the University of Florida Health System and contained 881 preeclamptic pregnancies from 2015 to 2021. The Integrated Data Repository managed the deidentification and transfer of patient data to the researchers.

### Feature Extraction and Preprocessing

The electronic medical records include medical history, obstetric diagnostic codes entered during each unique pregnancy, demographics, medications, laboratory results, and vital signs (Table S2 in Multimedia Appendix 1). The baseline model initially used age at the start of pregnancy, race, pregnancy start date, date of the first preeclampsia diagnosis, gravidity, parity, and previous history of preeclampsia at the trimester it was diagnosed. In addition, medical histories based on *ICD-10-CM* diagnosis codes were extracted using the Elixhauser Comorbidities definitions [8]. Current diagnoses entered within 20 weeks of gestation were extracted using the same *ICD-10-CM* diagnosis codes and definitions.

The full model includes all features in the baseline model. In addition, laboratory results, vital signs, and medications ordered before 20 weeks of gestation were also added. Laboratory tests that included a complete blood count were considered (Table S2 in Multimedia Appendix 1). Vital signs included diastolic and systolic BP. Laboratory findings and vitals collected from the start of pregnancy (0 days gestation) to 20 weeks (140 days) gestation were included. The mean, maximum, minimum, and SD for each laboratory value were calculated. Medication records were retrieved based on previous reports that medications prescribed during pregnancy may be related to preeclampsia development [9]. Patients who did not have any laboratory finding or vital data collected and entered in the EMR system within the first 20 weeks (~15%) were assigned as “missing”. These missing values were imputed using the predictive mean matching algorithm from the R package “mice” [10], which has been shown to produce the least-biased results for data sets that use feature selection [11-13]. The standards for missing data used for multiple imputations were followed, and imputation was performed on only the variables with no more than 20% missingness [14]. All numeric variables were log-transformed to adjust for skewness. Each feature in the medical history, clinical diagnosis, and medication categories was computed as a binary category: 1 for presence, and 0 for absence, to reduce feature dimensionality and improve interpretability. All analysis was conducted using R (version 4.2.2; The R Foundation) [15]. Data cleaning was carried out using the packages “dplyr” [16] and “gtsummary” [17].

### Feature Selection, Model Construction, and Evaluations

The UM discovery data set was randomly divided into a training set (80%) and a hold-out testing set (20%) after multiple imputations on missing variables. A Cox-proportional hazards model with Least Absolute Shrinkage and Selection Operator (LASSO) regularization was conducted through 5-fold cross-validation, using the “glmnet” [18] package in R. We used cross-validation to select the optimal LASSO hyperparameter (lambda) that gave the smallest mean squared error and then performed bootstrapping with 1000 replicates to calculate a concordance index (C-index) and 95% CIs for each data set (training, testing, and validation). The baseline model had an optimal lambda of 0.0058 (Figure S1A in Multimedia Appendix 2) and the full model had an optimal lambda of 0.0066 (Figure S1B in Multimedia Appendix 2). The baseline model had 31 features and the full model had 92 features before selection. Following regularized feature selection using the LASSO method on the training data sets, both final models have 5 selected features. The output of the Cox-PH model is the log hazard ratio, also called the prognosis index (PI), which depicts the relative risk of a patient when compared with the baseline hazard of the population. The full model was constructed in the same way as the baseline model.

External validation on each finalized model (baseline and full models) was done through collaboration with the University of Florida (UF), where the electronic health record (EHR) data and patient characteristics are different. Each feature chosen by the model was able to be identified in the UF validation cohort except for the nonsteroidal anti-inflammatory drug (NSAID) medication prescription, which was not available at the time of collection.

The performance of each model was evaluated using the C-index with bootstrapping of 1000 replicates to calculate 95% CI and *P* values from log-rank tests. The C-index is a metric to compare the discriminative power of a risk prediction model that describes the frequency of concordant pairs among all pairs of patients included in the model construction [19]. We used the C-index calculated from the “cindex” [20] function. Low- and high-risk pregnancies were stratified based on the median PI score of the model, and Kaplan-Meier curves were plotted for each risk group. Their differences were tested with log-rank tests using the training data set, hold-out testing data set, and the validation data set separately to evaluate the discriminative power of the model. The log-rank test is a significance test in survival analysis, with the null hypothesis that 2 groups have identical distributions of survival time. Any log-rank *P* value below .05 is considered statistically significant in these analyses. Feature importance was evaluated in the Cox-PH model by their HR *P* values. HR describes the relative contribution of a feature to the patient’s PI. In the context of our model, HRs above 1 shorten the gestational age of preeclampsia diagnosis, while HRs below 1 lengthen it.

We further measured model performance by calculating the sensitivity and specificity for each model, classified by predicting preeclampsia diagnosis by 34 and 37 weeks, respectively. We also plotted the area under the curve (AUC) from each testing data set for both models at both time points, using the “pROC” [21] package in R.

### Ethical Considerations

The institutional review board (IRB) of the UM Medical School (HUM#00168171) and the UF (#201601899) approved the original data collection and the use of the discovery cohort. All authors have permission for the use of this data. IRB approval was not required for the secondary analysis presented here, as it was deemed exempt. [22].

## Results

### Study Design and Data Set Overviews

**Figure 1:**
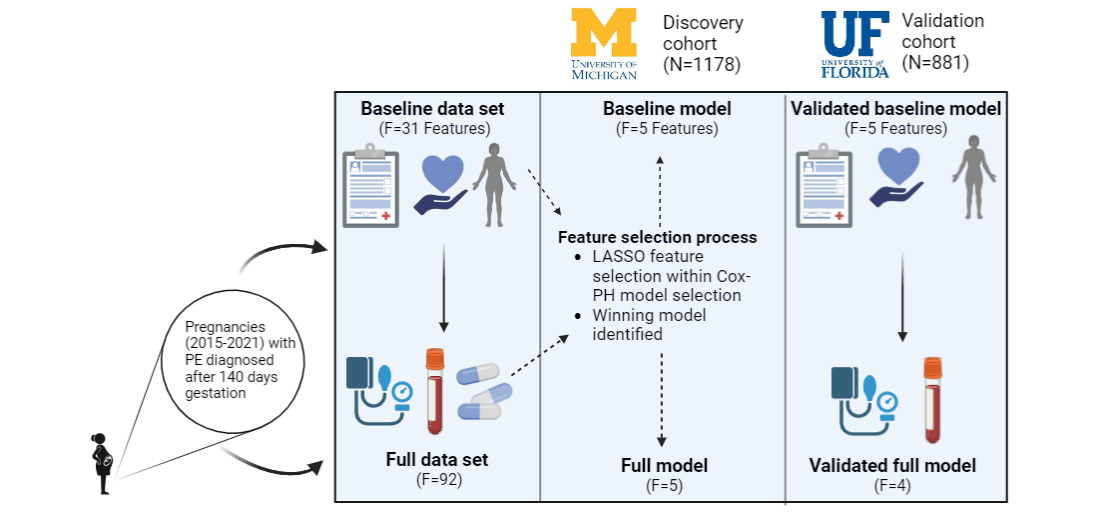
Study design and workflow for the University of Michigan preeclampsia cohort (N=1178) and the University of Florida preeclampsia cohort (N=881), 2015-2021. The discovery cohort was obtained from the University of Michigan Health System and a validation cohort of similar size and time was obtained from the University of Florida Health System. We constructed 2 preeclampsia predictive models: baseline and full model. The input variables in baseline models include patients’ demographics, lifestyle, comorbidities, and medical history (n=31) which were reduced to 5 features. The input for the full model includes additional lab tests and vital signs around preeclampsia diagnosis time, in addition to the variables in the baseline models (n=92), and was reduced to 5 features for the discovery cohort, and 4 features for the validation cohort. We trained the Cox-proportional hazards models with the Least Absolute Shrinkage and Selection Operator regularization, using 80% training from the University of Michigan discovery cohort. We tested it on 20% hold-out data from the same discovery cohort and validated it using the University of Florida validation cohort. Cox-PH: Cox proportional-hazard; LASSO: Least Absolute Shrinkage and Selection Operator; PE: preeclampsia; UF: University of Florida; UM: University of Michigan.

The overall study design is shown in Figure 1. The discovery cohort was extracted from patient records in the UM Health System from 2015 to 2022 with *ICD-10* (*International Statistical Classification of Diseases, Tenth Revision*) code access. All patients with a preeclampsia diagnosis after 20 weeks of gestation were included in the cohort, and other exclusion criteria are detailed in the Methods section. The finalized UM discovery cohort consists of EMRs from 1178 pregnancies. Using the same inclusion and exclusion criteria, 881 pregnancies were identified in the validation data set from UF. The patient characteristics for each cohort are listed in Table 1. The average maternal age was 30.2 years (SD 5.67) in the discovery cohort and 29.1 years (SD 6.18) in the validation cohort. The mean gestational age of preeclampsia onset was 251 (SD 25.4) days for the discovery cohort and 257 (SD 25.9) days for the validation cohort. We constructed and validated 2 models using this data: (1) a baseline model using only patient medical history, demographics, and diagnoses of any new medical issues within the first 20 weeks of gestation; and (2) a full model including those features from the baseline model, as well as additional information on medication, laboratory findings, and vitals within the first 20 weeks of pregnancy.

**Table 1:** Summaries of the characteristics of the University of Michigan preeclampsia discovery cohort (N=1178) and University of Florida preeclampsia validation cohort (N=881) for patients admitted from 2015 to 2021. Data are presented as the average (SD) or counts (% in the cohort).

Characteristics		Discovery cohort (N=1178)	Validation cohort (N=881)
Maternal age (years), mean (SD)		30.2 (5.67)	29.1 (6.18)
Gravidity, mean (SD)		2.31 (1.74)	2.82 (2.04)
Parity, mean (SD)		0.68 (1.12)	1.17 (1.5)
Number of fetuses, mean (SD)		1.07 (0.26)	1.04 (0.22)
Gestational age at PE onset (days), mean (SD)		251 (25.4)	257 (25.9)
Current smoker, n (%)		61 (5)	112 (13)
Current alcohol user, n (%)		311 (26)	184 (21)
**Race or ethnicity, n (%)**			
	African American	195 (17)	335 (38)
	Asian	74 (6)	19 (2)
	Hispanic	58 (5)	4 (1)
	History of PE^a^	184 (16)	117 (13)
	History of PE diagnosed in the second trimester	66 (6)	3 (<1)
**Medical history, n (%)**			
	Uncomplicated type I diabetes	34 (3)	19 (2)
	Uncomplicated type II diabetes	62 (5)	22 (3)
	Uncomplicated hypertension	201 (17)	81 (9)
	Kidney disease	14 (1)	1 (<1)
**Other clinical diagnoses within 20 weeks of gestation, n (%)**			
	Depression	265 (22)	19 (2)
	Mood and anxiety disorder	318 (27)	0

^a^PE: preeclampsia.

### Baseline Model

A baseline model was first built using medical history, demographics, and *ICD-10-CM* diagnosis codes of new medical conditions entered during the first 20 weeks of pregnancy. To build and test the model, we randomly split the data into an 80:20 ratio for training and testing data sets, and the Cox-PH model with LASSO (L1) regularization was built with the UM training data under 5-fold cross-validation. Alternatively, we explored ElasticNet (combined L1 and L2 regularization) as well as L2 penalization. However, the LASSO (L1) model overall performs better with higher C-indices and fewer features over these alternatives. We therefore chose LASSO as the regularization method (Table S3 in Multimedia Appendix 1).

We then applied this model to the 20% UM hold-out testing data and external UF validation cohort. The C-indices for the training, hold-out testing, and external validation data of the baseline model are 0.62 (95% CI 0.61-0.63), 0.64 (95% CI 0.60-0.65), and 0.61 (95% CI 0.59-0.63), confirming its validity. Table 2 shows the baseline model’s C-index and corresponding 95% CI values for each data set. To further facilitate interpretation, we classified each preeclampsia diagnosis prediction by the timeline of its occurrence, specifically by gestational weeks 34 and 37, using the UM hold-out testing data set. Such simple binary classification shows a sensitivity of 0.74, specificity of 0.50, and AUC of 0.65 for preeclampsia diagnosed at 34 weeks (Table 2). It has improved performance for preeclampsia diagnosis by 37 weeks, with a sensitivity of 0.82, specificity of 0.50, and AUC of 0.69 (Table 2 and Multimedia Appendix 3).

**Figure 2:**
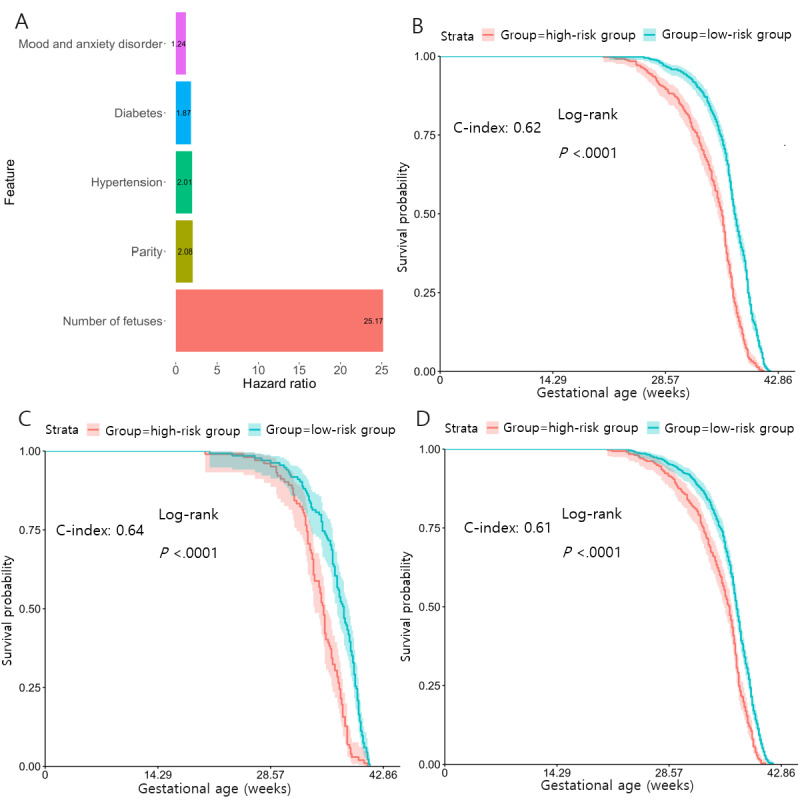
Gestational age of preeclampsia diagnosis baseline model features and performance. (A) Bar plot of hazard ratios of the selected features by Cox-proportional hazards method with Least Absolute Shrinkage and Selection Operator regularization. Ranging from smallest to largest hazard ratio: mood and anxiety disorder, diabetes, hypertension, parity, and number of fetuses. (B-D) Kaplan-Meier survival curves of high-risk (red) and low-risk (blue) pregnancies in the respective data sets, each with a log-rank test *P* value <.001. (B) University of Michigan training data set with a C-index of 0.62. (C) Hold-out testing set with a C-index of 0.64. (D) University of Florida validation data set with a C-index of 0.61.

Five features were selected for the baseline model. Their respective HRs and rankings in the multivariate Cox-PH are depicted in Figure 2A and Table 3. By the descending order of HR, these features are the number of fetuses in pregnancy of interest (HR 25.2; *P*<.001), parity (HR 2.08; *P*<.001), history of uncomplicated hypertension (HR 2.01; *P*<.001), history of uncomplicated type II diabetes (HR 1.87; *P*<.001), and a mood or anxiety disorder (HR 1.24; *P*=.01). All features increase preeclampsia risk and shorten the gestational age of preeclampsia diagnosis.

**Table 2:** Binarized performance for baseline and full models using 34- and 37-week preeclampsia diagnosis occurrences, measured using the hold-out testing data obtained from the randomly selected 20% patients from the University of Michigan discovery cohort.

Model version	34 weeks			37 weeks			
Metrics	Sensitivity	Specificity	AUC		Sensitivity	Specificity	AUC
Baseline	0.74	0.50	0.65		0.82	0.50	0.70
Full	0.98	0.51	0.70		0.86	0.50	0.70

**Table 3:** The selected features in the baseline model to predict the gestational age of preeclampsia diagnosis.

Features	Hazard ratio (95% CI)	*P* value
Number of fetuses	25.2 (10.7-59.4)	<.001
Parity	2.08 (1.54-2.81)	<.001
History of uncomplicated hypertension	2.01 (1.68-2.40)	<.001
History of uncomplicated type II diabetes	1.87 (1.41-2.49)	<.001
Mood and anxiety disorder	1.24 (1.07-1.43)	.01

To evaluate the discriminative power of this model, patients from the training data set were dichotomized into high- and low-risk groups by stratifying the samples using the median of the predicted PI (PI=1.17) from the model. The 2 risk groups showed significant differences in prognosis (Figure 2B and Table S4 in Multimedia Appendix 1). The high-risk group was characterized by higher parity and number of fetuses, while the low-risk pregnancies had no prevalence of hypertension (*P*<.001) or diabetes (*P*<.001). The median PI value above was applied to categorize samples into high versus low-risk groups in the hold-out (PI=1.17) and validation data (PI=2.38), similar to others [23-25]. As shown in Figures 2C and 2D, the KM curves on these 2 risk groups are also significantly different (*P*<.001).

### Full Model

**Figure 3:**
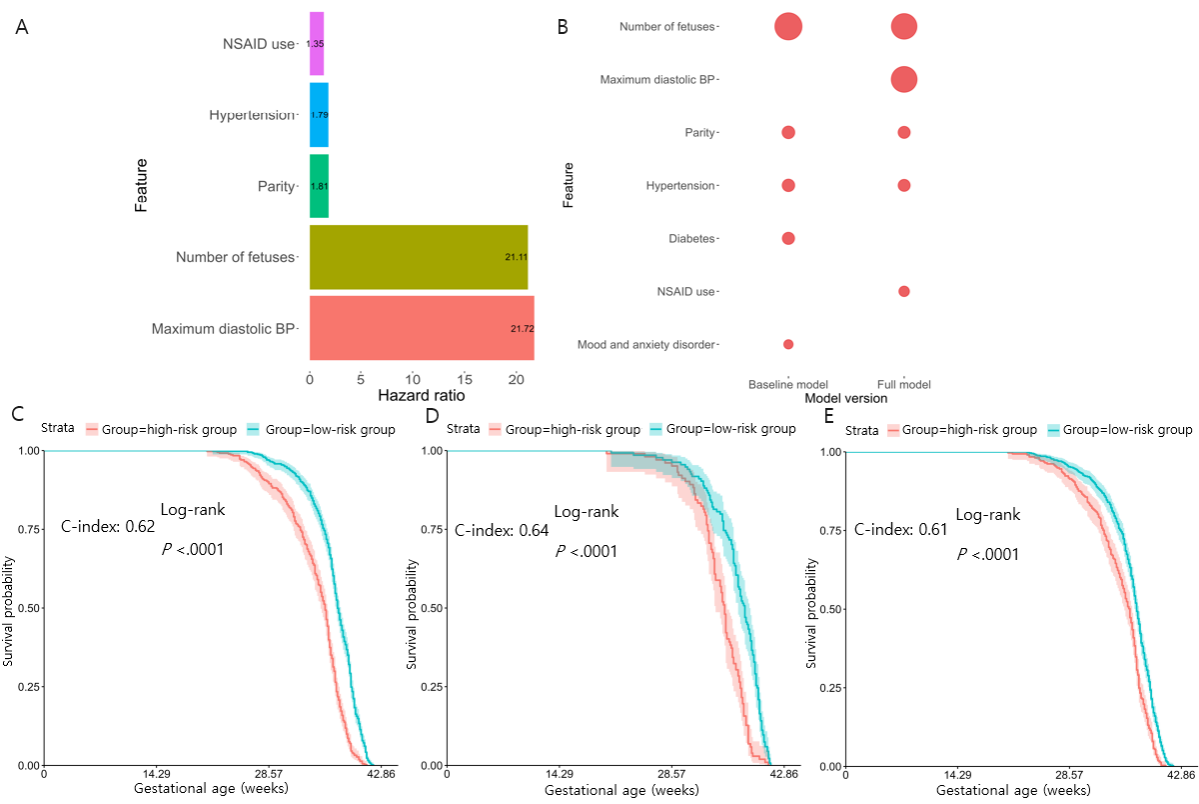
Gestational age of preeclampsia diagnosis full model features and performance. (A) Bar plot of hazard ratios of the selected features in the full model by Cox-proportional hazards method with Least Absolute Shrinkage and Selection Operator regularization. Ranging from smallest to largest hazard ratio: nonsteroidal anti-inflammatory drug use, hypertension, parity, number of fetuses, and maximum diastolic blood pressure. (B) The bubble plot of significant features from preeclampsia baseline and full models. The size of the bubbles represents the hazard ratio of each feature. The number of fetuses, parity, and hypertension were shared between both models with similar hazard ratios. (C-E) Kaplan-Meier survival curves of high-risk (red) and low-risk (blue) pregnancies in the respective data sets, each with a log-rank test *P* value <.001. (C) University of Michigan training data set with a concordance index of 0.62. (D) Hold-out testing set with a concordance index of 0.64. (E) University of Florida validation data set with a concordance index of 0.61. BP: blood pressure.

We next evaluated a model with the addition of laboratory findings, vitals, and medications prescribed in the first 20 weeks of gestation to the clinical data used in the baseline model. We constructed the new Cox-PH model, or the “full model,” in the same manner as the baseline model and obtained a 5-feature Cox-PH model (Figure 3A). Similar to the baseline model, LASSO regularization shows better overall performance than ElasticNet and L2 regularization and is chosen as the default (Table S3 in Multimedia Appendix 1). This new model reaches the C-indices of 0.66 (95% CI 0.64-0.67) and 0.69 (95% CI 0.64-0.70) for the training and hold-out testing data sets, respectively. It also yields a C-index of 0.61 (95% CI 0.60-0.63) on the UF validation cohort, despite missing 1 feature (NSAID medication) in the UF cohort. Table 2 lists the full-model C-indices and 95% CIs for each data set. Similar to the baseline model, to help interpretation, we classified each preeclampsia diagnosis prediction using the timeline of preeclampsia occurrence by gestational weeks 34 and 37, respectively, using the UM hold-out testing data set. It yields a sensitivity of 0.98, specificity of 0.51, and AUC of 0.70 for correctly predicting preeclampsia by week 34 (Table 2). The model has an improved correct diagnosis by week 37, with a sensitivity of 0.86, specificity of 0.50, and AUC of 0.70 (Table 2 and Multimedia Appendix 3).

The full model also yields 5 features, all with positive HRs (Figure 3A and Table 4). In descending order of HR, these features are maximum diastolic blood pressure (HR 21.7; *P*<.001), number of fetuses in current pregnancy (HR 21.1; *P*<.001), parity (HR 1.81; *P*<.001), history of uncomplicated hypertension (HR 1.79; *P*<.001), and NSAID medication prescription (HR 1.35; *P*<.001). Three of these features, namely the number of fetuses, history of uncomplicated hypertension, and parity features were also selected by the baseline model (Figure 3B). Table S5 in Multimedia Appendix 1 shows each of the features and their HRs in a univariate analysis. Their HRs across the baseline and full models remain very similar and had *P* values less than .05, suggesting that they are all significant in predicting preeclampsia onset time regardless of the other additional input information. Maximum diastolic BP and NSAID medication prescription are newly selected features unique to the full model (Figures 3A and 3B).

Like the baseline model, we stratified patients into high- versus low-risk groups using the median predicted PI value of 5.15 from the training data set (Figure 3C). The high-risk group was characterized by higher parity, a higher number of fetuses, and higher maximum diastolic BP (Table S4 in Multimedia Appendix 1). In contrast, the low-risk group had no history of hypertension and rare use of NSAID medication. BP had the most statistically significant difference (*P*<.001), as expected. The same median threshold was applied to the 20% hold-out testing data set (PI=5.08) and validation data (PI=5.18) for dichotomization (Figures 3D and 3E). KM curves on these 2 risk groups in the testing set have even more significant differences in their gestational age at diagnosis (*P*<.001). Both models are to be used by entering patient information in the predictors to predict when the patient may develop preeclampsia.

To determine the potential impact of missing data on modeling results, we explored building a baseline and full model with only cases that had complete BP data—the main selected feature in the full model. Table S6 in Multimedia Appendix 1 shows the selected features of both of these models. The complete cases baseline model had a training C-index of 0.63 and a testing C-index of 0.64. The complete cases full model had a training C-index of 0.67 and a testing C-index of 0.65. Due to similar performance and selected features, it can be safely assumed that imputation had little impact on the finalized models.

**Table 4:** Summary of the selected features in the full model to predict the gestational age upon preeclampsia diagnosis.

Features	Hazard ratio (95% CI)	*P* value
Maximum diastolic blood pressure	21.7 (7.93-59.8)	<.001
Number of fetuses	21.1 (9.88-45.1)	<.001
Parity	1.81 (1.37-2.39)	<.001
History of uncomplicated hypertension	1.79 (1.53-2.11)	<.001
NSAID^a^ medication	1.35 (1.15-1.58)	<.001

^a^NSAID: nonsteroidal anti-inflammatory drug.

## Discussion

### Principal Results

This paper is the first of its kind to implement and externally validate a prognosis-predicting model for preeclampsia onset time using EHR data from the first 20 weeks of pregnancy [26]. These models confirmed that factors such as BP in the first 20 weeks of pregnancy, the number of fetuses, parity, and previous history of hypertension are associated with earlier preeclampsia onset time. Moreover, comorbidities such as gestational diabetes and anxiety, as well as NSAID medication, shorten preeclampsia onset time. The similar performance across validation and development data sets provides confidence in the accuracy of the predictive outputs.

### Comparison With Previous Work

A recent study stratified patients with preeclampsia by gestational age to build classification models, resulting in many models that are difficult for clinicians to select from [27]. Moreover, these classification models cannot predict the gestational age of onset for an individual patient, thus failing to assist clinicians in making early decisions on delivery plans and proper antenatal care. Unlike most other accurate preeclampsia onset time prediction models, our models only use EMR data from the first 20 weeks of pregnancy and do not require advanced testing inputs, such as biomarkers [27], enabling earlier use in clinics. In a systematic review of 68 preeclampsia prediction models [27], only 6% (4/68) of them were externally validated, and those not requiring complex biomarker features had much lower AUCs (0.58-0.61) than the models presented here (AUC 0.65-0.70), highlighting the accuracy of our models once validated against a different patient population.

### Clinical and Research Implications

Due to the difficulty in predicting preeclampsia, accurate models that can identify women at high risk for preeclampsia can provide early targeted treatment as well as increased surveillance to reduce adverse outcomes [28]. The models here not only confirm the importance of some previously known risk factors, such as the number of fetuses, history of hypertension, and parity but also assign quantitative scores (weights) on the importance of these risk factors relative to each other. This is a significant advancement from most of the other studies focusing on a single risk factor. It also provides clinicians as well as pregnant women with quantitative tools to assess the onset time of preeclampsia more accurately, beyond the qualitative assessment of risks. Risk factors with higher weights can take a higher priority for clinicians to identify potential patients with preeclampsia. The fact that maximum diastolic BP had the highest HR in the full model confirms the importance of monitoring BP as early as possible, even before preeclampsia is diagnosed clinically [29]. More importantly, it identifies additional alarming factors to be considered in predicting preeclampsia diagnosis at gestational age, such as mood and anxiety disorder.

Further risk stratification of the survival models had slightly low specificity values in predicting the dichotomous diagnosis of preeclampsia at 34 and 37 weeks, suggesting that the continuous risk diagnosis has overall better performance compared with the simple binary prediction. However, the stratification may offer an easier way to identify women who may benefit more significantly from prevention therapy and need more medical attention from doctors for the possibility of preeclampsia. EHR-based models can serve as a screening test. For the patients that are potentially false positive for preeclampsia due to the lower specificity of the model, additional confirmative diagnostic tests using very specific biomarkers should be done, as practiced clinically.

Earlier studies using all pregnant women also revealed that mood and anxiety disorders increase the risk of preeclampsia [30]. We further show that within patients with preeclampsia, mood and anxiety disorders shorten the onset time of preeclampsia. This provides more context for clinicians to identify pregnant patients who present mood and anxiety disorders and provide preventative care to reduce preeclampsia onset risk. The molecular mechanism linking mood and anxiety disorders with preeclampsia is worth further research. We also show that NSAID use is positively associated with earlier onset of preeclampsia. However, aspirin is a common NSAID used by pregnant women at risk for preeclampsia early in pregnancy [31]. It was suggested that NSAID use may serve as a proxy for the interaction of many unmeasured risk factors [32]. Thus, the positive association of NSAID to the earlier onset of preeclampsia may indicate that it is a marker of high-risk preeclampsia in the population, rather than the cause of it.

### Strengths and Limitations

A particular strength of the models here is their simplicity despite being quantitative. The models can also be generalized to different medical centers and hospitals, given the good accuracy when validated by vastly different institutions with different protocols, data collection, and data storage. There is a growing need for evidence-based and effective tools for clinicians to screen women at high risk of preeclampsia early in pregnancy, in the first and early second trimesters. This model supplies this need for early prediction models that previous models have not been able to fulfill [33]. Most clinical models recently published include many predictors from biomarkers and ultrasound markers that need special procedures [34], further suggesting that a simpler model on routinely collected clinical data is valuable to be implemented in a clinical setting. The main strength of this modeling for clinical use proposed here is providing more context in screening patients at risk for preeclampsia.

Our ultimate goal is to implement these models in the health care system, for example, starting from the University of Michigan. Potential challenges for implementing these models in a clinical setting include institutional buy-in, installation of the software in a HIPAA (Health Insurance Portability and Accountability Act)-compliant computing environment, and explaining the meaning of risk factors and model results to patients informatively without overly stressing them. In addition, these models may potentially require more active updating for improving accuracy, by considering additional multicenter data. Also, the current Cox-PH model is not designed to include longitudinal observations, limiting the kind of input variables to be incorporated into the model. Future work may benefit from more sophisticated modeling approaches [35]. Besides EHR, other omics information such as genetics, genomics, proteomics, and metabolomics using maternal blood samples [34] may be used, if they are available, to improve the model performance. However, implementing multimodal and complex models like this in the clinical setting is additionally challenging and would require more advanced modeling that can calculate individual risk scores for clinical application. It is also important to note the use of EHR data to extract medication prescriptions does not accurately capture the actual use or adherence of the medication by patients, and future research could be strengthened by combining data sources that provide such information.

### Conclusions

In conclusion, this study reports prognosis models to predict the onset gestational age of preeclampsia with EMR data before the first 20 weeks of pregnancy. They identify clinical and physiological factors that clinicians should monitor as indicators of early preeclampsia development.

## Appendix

Multimedia Appendix 1 Supplementary tables.

Multimedia Appendix 2 Lambdas from Least Absolute Shrinkage and Selection Operator (LASSO) regularization from the baseline and full preeclampsia (PE) prediction models. (A) Scatterplot of tested lambda values and associated errors from baseline model LASSO regularization. (B) Scatterplot of tested lambda values and associated errors from full model LASSO regularization.

Multimedia Appendix 3 AUC values of preeclampsia (PE) diagnosed at 34 and 37 weeks for the baseline and full PE prediction models. (A) Plot of the sensitivity, specificity, and area under the curve (AUC) values for the baseline model of predicting PE diagnosed at 34 weeks (red, AUC=0.654) and 37 weeks (green, AUC=0.694) for the testing dataset. (B) Plot of the sensitivity, specificity, and AUC values for the full model of predicting PE diagnosed at 34 weeks (red, AUC=0.697) and 37 weeks (green, AUC=0.700) for the testing data set.

## Abbreviations

AUC: area under the curve
BP: blood pressure
C-index: concordance index
EHR: electronic health record
EMR: electronic medical record
HIPAA: Health Insurance Portability and Accountability Act
HR: hazard ratio
ICD-10: International Statistical Classification of Diseases, Tenth Revision
ICD-10-CM: International Classification of Diseases, Tenth Revision, Clinical Modification
IRB: institutional review board
LASSO: Least Absolute Shrinkage and Selection Operator
NSAID: nonsteroidal anti-inflammatory drug
PI: prognosis index
UF: University of Florida
UM: University of Michigan

## Data Availability

The data sets generated during and/or analyzed during this study are not publicly available due to the presence of patient-protected health information. Data are available upon reasonable request and must be submitted on an individual basis to the home institution. Table S2 in Multimedia Appendix 1 lists all the EHR features extracted from the UM system that were considered in the starting model.
