# Supplementary material for: Five-Feature Models to Predict Preeclampsia Onset Time From Electronic Health Record Data: Development and Validation Study
Source: J Med Internet Res. 2024 Aug 14;26:e48997. doi: 10.2196/48997 (PMC11358663; doi:10.2196/48997)
Supplement: Multimedia Appendix 1 [file jmir_v26i1e48997_app1.docx]

*Supplementary Tables*

*Supplementary Table 1: Inclusion/Exclusion Criteria for the University of Michigan and University of Florida EHR*

| Medical Condition | Inclusion/Exclusion | ICD Codes |
| --- | --- | --- |
| HELLP syndrome | Exclusion | O14.2* |
| Pre-existing hypertension with superimposed preeclampsia | Exclusion | O11* |
| Postpartum preeclampsia | Exclusion | O14.95 |
| Preeclampsia | Inclusion | O14.0*, O14.1*, O14.9* |

*Supplementary Table 2: All features extracted from EMR to build the University of Michigan discovery cohort and the University of Florida validation cohort*

| Baseline Features | | Additional Features | |
| --- | --- | --- | --- |
| *Maternal Information* | *Medical History* | *Lab Tests* | *Vitals* |
| Gravidity | Uncomplicated primary hypertension | Mean platelet Count | Mean diastolic BP |
| Parity | Uncomplicated type II diabetes | Minimum platelet count | Minimum diastolic BP |
| Number of fetuses | Complicated type II diabetes | Maximum platelet count | Maximum diastolic BP |
| Gestational age at diagnosis (days) | Uncomplicated type I diabetes | Standard deviation platelet count | Standard deviation diastolic BP |
| In vitro fertilization | Complicated type I diabetes | Mean hematocrit | Mean systolic BP |
| Maternal age | Systemic lupus erythematosus | Minimum hematocrit | Minimum systolic BP |
| *Demographics* | Chronic kidney disease | Maximum hematocrit | Maximum systolic BP |
| African American race | Polycystic ovarian syndrome | Standard deviation hematocrit | Standard deviation BP |
| Asian race | Autoimmune disorders | Mean hemoglobin | Medication |
| Hispanic ethnicity | Comorbidities | Minimum hemoglobin | Acetaminophen |
| PE history | Unspecified mood or anxiety disorder | Maximum hemoglobin | Benzodiazepines |
| Past PE | Depression | Standard deviation Hemoglobin | Triptans |
| Past severe PE | Sleep apnea | Mean MCH | Antacids |
| Past PE diagnosed in second trimester | Infection | Minimum MCH | Diuretics |
| Past PE diagnosed in third trimester | BMI over 35 | Maximum MCH | Calcium channel blockers |
|  | Headaches | Standard Deviation MCH | Angiotensin-converting-enzyme Inhibitors |
|  | New autoimmune disorder | Mean MCV | Antidepressants |
|  | Alcohol use | Minimum MCV | Allergy medication |
|  | Drug use | Maximum MCV | Antibiotics |
|  | Smoking status | Standard deviation MCV | Nasal sprays |
|  |  |  |  |
|  |  | Mean RBCC | Thyroid medication |
|  |  | Minimum RBCC | Yeast infection treatment |
|  |  | Maximum RBCC | NSAIDs |
|  |  | Standard deviation RBCC | Sedatives |
|  |  | Mean RCDW |  |
|  |  | Minimum RCDW |  |
|  |  | Maximum RCDW |  |
|  |  | Standard Deviation RCDW |  |
|  |  | Mean WBCC |  |
|  |  | Minimum WBCC |  |
|  |  | Maximum WBCC |  |
|  |  | Standard Deviation WBCC |  |
|  |  | Mean MCHC |  |
|  |  | Minimum MCHC |  |
|  |  | Minimum MCHC |  |
|  |  | Standard Deviation MCHC |  |
|  |  | Mean MPV |  |
|  |  | Minimum MPV |  |
|  |  | Maximum MPV |  |
|  |  | Standard Deviation MPV |  |

*Supplementary Table 3: Comparison of LASSO (L1), ElasticNet (L1+L2) and L2 penalty methods.*

| Penalty | Model | Feature | HR and 95% CI | Training C-Index | Testing C-Index |
| --- | --- | --- | --- | --- | --- |
| L1+L2 | Baseline | Number of fetuses | 11.1(6.22, 20.0) | 0.64 | 0.62 |
|  |  | History of uncomplicated type II diabetes | 1.82(1.40, 2.37) |  |  |
|  |  | History of uncomplicated hypertension | 2.01(1.72, 2.36) |  |  |
|  |  | Gravidity | 1.31(0.92, 1.87) |  |  |
|  |  | Parity | 1.28(1.02, 1.61) |  |  |
|  | Full | Max diastolic BP | 14.8(6.23, 34.9) | 0.67 | 0.64 |
|  |  | Number of fetuses | 10.4(5.78, 18.67) |  |  |
|  |  | History of uncomplicated hypertension | 1.79(1.52, 2.10) |  |  |
|  |  | NSAID medication | 1.33(1.13, 1.56) |  |  |
|  |  | Gravidity | 1.30(0.912, 1.85) |  |  |
|  |  | Parity | 1.18(0.946, 1.49) |  |  |
| L2 | Baseline | Number of fetuses | 10.3(5.77, 18.4) | 0.63 | 0.65 |
|  |  | History of uncomplicated hypertension | 2.08(1.78, 2.44) |  |  |
|  |  | Parity | 1.49(1.30, 1.71) |  |  |
|  |  | Depression | 1.35(1.17, 1.55) |  |  |
|  | Full | Number of fetuses | 10.5(5.85, 18.9) | 0.65 | 0.66 |
|  |  | Mean diastolic BP | 6.56(0.99, 43.3) |  |  |
|  |  | Maximum diastolic BP | 2.81(0.54, 14.7) |  |  |
|  |  | Maximum systolic BP | 2.70(0.70, 10.4) |  |  |
|  |  | History of uncomplicated hypertension | 1.75(1.49, 2.06) |  |  |
|  |  | NSAID medication | 1.36(1.15, 1.60) |  |  |
|  |  | History of uncomplicated hypertension | 1.75(1.49, 2.06) |  |  |
|  |  | Parity | 1.34(1.16, 1.55) |  |  |
| L1 | Baseline | Number of fetuses | 25.2(10.7, 59.4) | 0.62 | 0.64 |
|  |  | Parity | 2.08(1.54, 2.81) |  |  |
|  |  | History of uncomplicated hypertension | 2.01(1.68, 2.40) |  |  |
|  |  | History of uncomplicated type II diabetes | 1.87(1.41, 2.49) |  |  |
|  |  | Mood/anxiety disorder | 1.24(1.07, 1.43) |  |  |
|  | Full | Max diastolic blood pressure | 21.7(7.93, 59.8) | 0.66 | 0.69 |
|  |  | Number of fetuses | 21.1(9.88, 45.1) |  |  |
|  |  | Parity | 1.81(1.37, 2.39) |  |  |
|  |  | History of uncomplicated hypertension | 1.79(1.53, 2.11) |  |  |
|  |  | NSAID medication | 1.35(1.15, 1.58) |  |  |

*Supplementary Table 4: Mean feature difference across risk groups for baseline and full model testing datasets*

| Model | Feature | Low-Risk | High-Risk | Welch Two-sample p-value |
| --- | --- | --- | --- | --- |
| Baseline | Parity | 1.14 | 2.11 | <0.001 |
|  | Number of fetuses | 1.00 | 1.13 | <0.001 |
|  | Mood/anxiety disorder | 0.21 | 0.36 | <0.001 |
|  | History of uncomplicated hypertension | 0.00 | 0.41 | <0.001 |
|  | History of uncomplicated type II diabetes | 0.00 | 0.13 | <0.001 |
| Full | Maximum diastolic BP | 71.3 | 82.8 | <0.001 |
|  | Parity | 1.13 | 1.82 | <0.001 |
|  | Number of fetuses | 1.00 | 1.10 | <0.001 |
|  | NSAID medication | 0.01 | 0.39 | <0.001 |
|  | History of uncomplicated Hypertension | 0.00 | 0.34 | <0.001 |

*Supplementary Table 5: Univariate analysis of selected features*

| Feature | Hazard Ratio | 95% CI | p-value |
| --- | --- | --- | --- |
| Maximum diastolic BP | 55.7 | 24.9, 124 | <2e-16 |
| Number of fetuses | 10.5 | 5.84, 18.7 | 2e-12 |
| History of uncomplicated hypertension | 2.18 | 1.86, 2.54 | <2e-16 |
| History of uncomplicated type II diabetes | 2.10 | 1.62, 2.71 | 4e-07 |
| NSAID medication | 1.90 | 1.64, 2.20 | 6e-16 |
| Parity | 1.68 | 1.48, 1.92 | 1e-13 |
| Mood/anxiety disorder | 1.30 | 1.14, 1.48 | 1e-04 |

*Supplementary Table 6: Selected features and HRs for complete cases full model*

| Feature | Hazard Ratio | 95% CI |
| --- | --- | --- |
| Number of fetuses | 2.26 | 1.73, 2.96 |
| History of uncomplicated hypertension | 1.69 | 1.40, 2.03 |
| History of uncomplicated type II diabetes | 1.58 | 1.17, 2.14 |
| NSAID medication | 1.42 | 1.19, 1.68 |
| Mood/anxiety disorder | 1.26 | 1.09, 1.45 |
| Parity | 1.09 | 1.03, 1.16 |
| Maximum diastolic BP | 1.02 | 1.01, 1.03 |
