# Supplementary figures and images for: Five-Feature Models to Predict Preeclampsia Onset Time From Electronic Health Record Data: Development and Validation Study
Source: J Med Internet Res. 2024 Aug 14;26:e48997. doi: 10.2196/48997 (PMC11358663; doi:10.2196/48997)

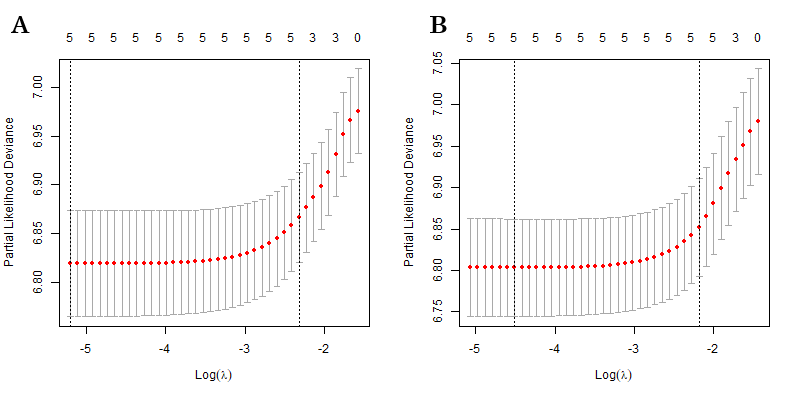

Supplement: Multimedia Appendix 2 [file jmir_v26i1e48997_app2.png]

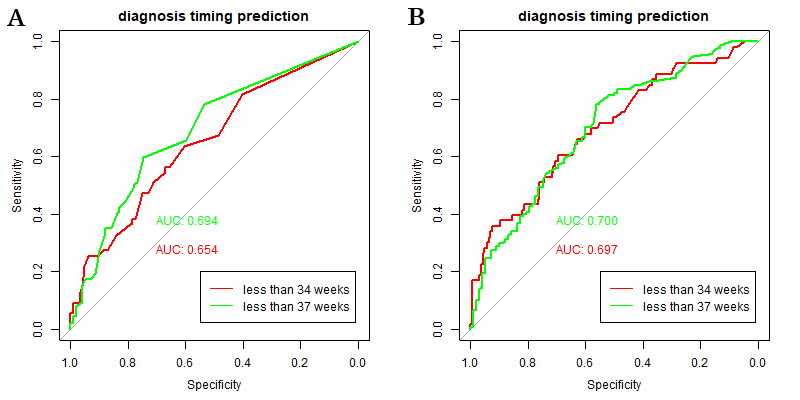

Supplement: Multimedia Appendix 3 [file jmir_v26i1e48997_app3.png]
